# Acute multi-sgRNA knockdown of KEOPS complex genes reproduces the microcephaly phenotype of the stable knockout zebrafish model

**DOI:** 10.1371/journal.pone.0191503

**Published:** 2018-01-18

**Authors:** Tilman Jobst-Schwan, Johanna Magdalena Schmidt, Ronen Schneider, Charlotte A. Hoogstraten, Jeremy F. P. Ullmann, David Schapiro, Amar J. Majmundar, Amy Kolb, Kaitlyn Eddy, Shirlee Shril, Daniela A. Braun, Annapurna Poduri, Friedhelm Hildebrandt

**Affiliations:** 1 Department of Medicine, Boston Children’s Hospital, Harvard Medical School, Boston, Massachusetts, United States of America; 2 Epilepsy Genetics Program and F.M. Kirby Neurobiology Center, Department of Neurology, Boston Children's Hospital, Harvard Medical School, Boston, Massachusetts, United States of America; National Institutes of Health, UNITED STATES

## Abstract

Until recently, morpholino oligonucleotides have been widely employed in zebrafish as an acute and efficient loss-of-function assay. However, off-target effects and reproducibility issues when compared to stable knockout lines have compromised their further use. Here we employed an acute CRISPR/Cas approach using multiple single guide RNAs targeting simultaneously different positions in two exemplar genes (*osgep* or *tprkb*) to increase the likelihood of generating mutations on both alleles in the injected F0 generation and to achieve a similar effect as morpholinos but with the reproducibility of stable lines. This multi single guide RNA approach resulted in median likelihoods for at least one mutation on each allele of >99% and sgRNA specific insertion/deletion profiles as revealed by deep-sequencing. Immunoblot showed a significant reduction for Osgep and Tprkb proteins. For both genes, the acute multi-sgRNA knockout recapitulated the microcephaly phenotype and reduction in survival that we observed previously in stable knockout lines, though milder in the acute multi-sgRNA knockout. Finally, we quantify the degree of mutagenesis by deep sequencing, and provide a mathematical model to quantitate the chance for a biallelic loss-of-function mutation. Our findings can be generalized to acute and stable CRISPR/Cas targeting for any zebrafish gene of interest.

## Introduction

For nearly two decades injection of morpholino oligonucleotides (MO) has been employed as a scalable “acute” gene loss-of-function assay in Xenopus [1] and zebrafish embryos [2]. MO are antisense oligonucleotides that bind to mRNA and facilitate gene function delineation in early development. They are injected in two to four cell stage embryos and block translation in general. Splice site specific MO enable the silencing of only zygotic mRNA to investigate the influence of maternal mRNA [3].

However, recently Kok et al. [4] demonstrated for more than 20 genes that there is a very poor correlation between morphant zebrafish phenotypes and phenotypes of stable mutant zebrafish lines. They found that approximately 80% of morphant phenotypes were not observed in mutant embryos. Therefore, the use of MO as an acute knock down (KD) approach has to be viewed critically and careful guidelines have been suggested [5]. Similar discrepant results have been identified when comparing shRNA KD versus CRISPR/Cas9 knock-out (KO) in cell culture [6]. One mechanism that contributes to the phenotypic discrepancy may be a genetic compensation that may occur in stable CRISPR/Cas KO zebrafish, but not in acute KD [7]. A stable mutant zebrafish line corresponds better to the situation in human patients with genomic mutations. However, an “acute” genomic KO/mutant approach allows for fast screening early on, and easily produces scalable numbers of KO larvae for large scale experiments such as chemical screens [8, 9].

Phenotype screening in the injected F0 generation using CRISPR/Cas9 has been described in zebrafish [10–12]. Different groups have used multiplexed sgRNAs to target different genes at the same time to screen for F0 phenotypes [10, 12, 13]. However, not every sgRNA target is suitable for F0 screening due to the mosaicism of wildtype alleles, in frame mutations and loss-of-function alleles [14]. Therefore a quantitative approach is needed to ensure that a lack of phenotype is not due to a lack of loss-of function alleles.

To compare resulting phenotypes for acute and stable KO, we employed two recently generated stable heterozygous zebrafish KO lines for two genes, *osgep* (NM_001017751) and *tprkb* (NM_001007373) that showed a robust microcephaly and survival phenotype [15]. We generated for these two genes an acute CRISPR/Cas9 approach using multiple guide RNAs (multi sgRNA) that target the same gene to maximize the initial KO and that attenuate the limitations of a mosaic genotype. Here we compare the resulting phenotypes for acute and stable KO, quantify the degree of mutagenesis by deep sequencing, calculate the chance for a biallelic loss-of-function mutation, and compare the phenotypic features of acute multi sgRNA KO to stable CRISPR/Cas9 mutant lines. Our findings can be generalized to acute and stable CRISPR/Cas targeting for virtually any zebrafish gene of interest.

## Material and methods

Zebrafish experiments were performed in *Danio rerio*, strain *l-fabp*: VDBP-GFP (AB). All national and institutional guidelines for the care and use of laboratory animals were followed. The zebrafish experiments were approved by the Boston Children’s Hospital (BCH) Institutional Animal Care and Use Committee (IACUC).

### Generation and phenotypic characterization of stable zebrafish KO lines by CRISPR/Cas9

#### Target selection and sgRNA generation

Single guide RNA (sgRNA) targets were selected using the CHOPCHOP online tool v1 (https://chopchop.rc.fas.harvard.edu [16]) following their ranking algorithm. The algorithm takes into account all potential off-target sites differing in up to 2 nucleotides, GC-content and presence of a guanine residue in the last position before the Protospacer Adjacent Motif (PAM) sequence since these factors influence the efficiency of sgRNA binding and Cas9 cleavage [17]. Targets were chosen in early exons to potentially introduce early frameshift mutations to maximize loss of function of the protein. sgRNAs were generated by *in-vitro* transcription from oligonucleotide based templates using the MEGAscript T7 Transcription Kit (Ambion) [17]. Since sgRNA activity is higher if two guanine bases follow the T7 promotor [17], template sequences were modified accordingly if necessary (S1 Table). The resulting change of one or two nucleotides in the 5’ end of the gRNA results in a higher indel frequency [17] and does not reduce specificity [18].

#### Microinjection, mutation analysis and breeding

2 μl of sgRNA stock (500 ng/μl) were mixed with 2 μl of recombinant Cas9 protein (1 μg/μl, PNA Bio, Thousand Oaks, CA) and incubated on ice for at least 10 min to allow formation of the sgRNA/Cas9 complex. 2 nl of the injection mix was injected intracellularly in one-cell stage zebrafish embryos using glass needles and a micromanipulator. DNA was extracted from 10 pooled injected embryos and an uninjected control group at 48 hours post fertilization (hpf) using the HotShot protocol [19]. Mutagenesis was determined by a T7 endonuclease assay as described before [20]. Positive clutches (F0 generation) were raised to adulthood and outcrossed against wildtype fish. Germline transmission was also determined by the T7 endonuclease assay. Positive clutches (F1 generation) were raised to adulthood and genotyped individually. Fish carrying the same mutation were pooled being the founders of the heterozygous stable knock out line.

For generation of the acute multi-sgRNA CRISPR KO, equal amounts of the sgRNAs stocks (500 ng/μl) were mixed and 2 μl of the mix was used as described above.

#### Survival analysis

Embryos were generated by timed breedings and kept in fish water containing 0.002% methylene blue until 24 hpf followed by fish water only. Larvae were transferred to rotifer feeding solution at 8 days post fertilization (dpf), the solution being changed every other day with additional daily rotifer feeding. The dishes were monitored twice a day for 22 dpf. The endpoint was reached when minimal residual cardiac activity without visible blood flow in the tail vein was observed during the first observation interval ranging from 1 dpf to 5 dpf. From 5 to 22 dpf, the additional humane endpoint was reached when larvae showed impaired swimming behavior and turned on their side instead of swimming upright. Larvae that reached the endpoint were euthanized immediately using Tricaine (0.4–0.8 mg/ml) to minimize distress. 1500 zebrafish larvae were included in the survival analysis. 50% of the larvae were found dead without meeting the endpoint criteria before. Death of control larvae between 9 and 12 dpf happened most likely due to insufficient transition to proper feeding behavior. Acute and stable *osgep* and *tprkb* KO larvae died most likely due to increased neuronal apoptosis [15].

For the stable CRISPR KO lines, the DNA was extracted individually from larvae that reached the endpoint and the genotype was confirmed by Sanger sequencing.

#### Microcephaly assay in Zebrafish

Zebrafish larvae were embedded in 1% ultra-low gelling temperature agarose (Type IX-A, Sigma-Aldrich, St. Louis, MO) and imaged under a stereomicroscope (Zeiss, Germany) from a dorsal view. Total body length and head diameter through the rear third of the eye lens were measured using Fiji ImageJ [21], the experimenter being blind towards the genotype. Head diameter to total body length ratio was calculated as”microcephaly index”. Significant differences were calculated using the one-way ANOVA test with multiple comparisons and a standard confidence interval of 95%.

### Deep sequencing

DNA from 96 individual larvae for each *osgep* and *tprkb* was extracted at 48 hpf using the HotShot protocol [19]. Primers were designed using UCSC genome browser data (http://genome.ucsc.edu/) [22] and the in-silico PCR tool on assembly Zv9/danRer7. Amplicon sizes ranged from 252 to 302 bp. Universal tags for the barcoding PCR were added to the primer sequences (S2 Table). All primers were purchased from Integrated DNA Technologies, Coralville, IA.

Initial genotyping PCR was performed using HotStarTaq Master Mix Kit (Quiagen, Hilden, Germany), each reaction containing 4 μl water, 1 μl DNA, 1 μl forward primer (10 nM), 1 μl reverse primer (10nM) and 6 μl Master Mix. A second unidirectional barcoding PCR was performed as described before [23] using Access Array Target Specific Primers (Fluidigm, San Francisco, CA) and the FastStart High Fidelity PCR system (Roche, Mannheim, Germany) according to the manufacturer’s instructions. Samples were pooled after barcoding and gel purified (270–350 bp) on a 1.5% agarose gel. Library DNA concentration was determined by Bioanalyzer (Agilent, Santa Clara, CA) and a 14 pM dilution of the library was sequenced on a Illumina MiSeq (Illumina, San Diego, CA) with v2 chemistry using a 500 cycles kit according to the manufacturer’s instructions. Deep sequencing data was analyzed using the tool CRISPResso [24].

### Statistical analysis

Statistical analysis was performed using Graph Pad Prism^®^ (version 7.00; GraphPad Software, Inc, La Jolla, CA). Significance was calculated using unpaired one-way ANOVA with multiple comparisons and a standard confidence interval of 95% for the microcephaly assay. *Post hoc* analysis was performed according to Tukey.

Survival curves were analyzed by Log-rank (Mantel-Cox) test.

### Densitometry analysis of immunoblots

Densitometry analysis was performed using Fiji ImageJ [21].

## Results

**Fig 1:**
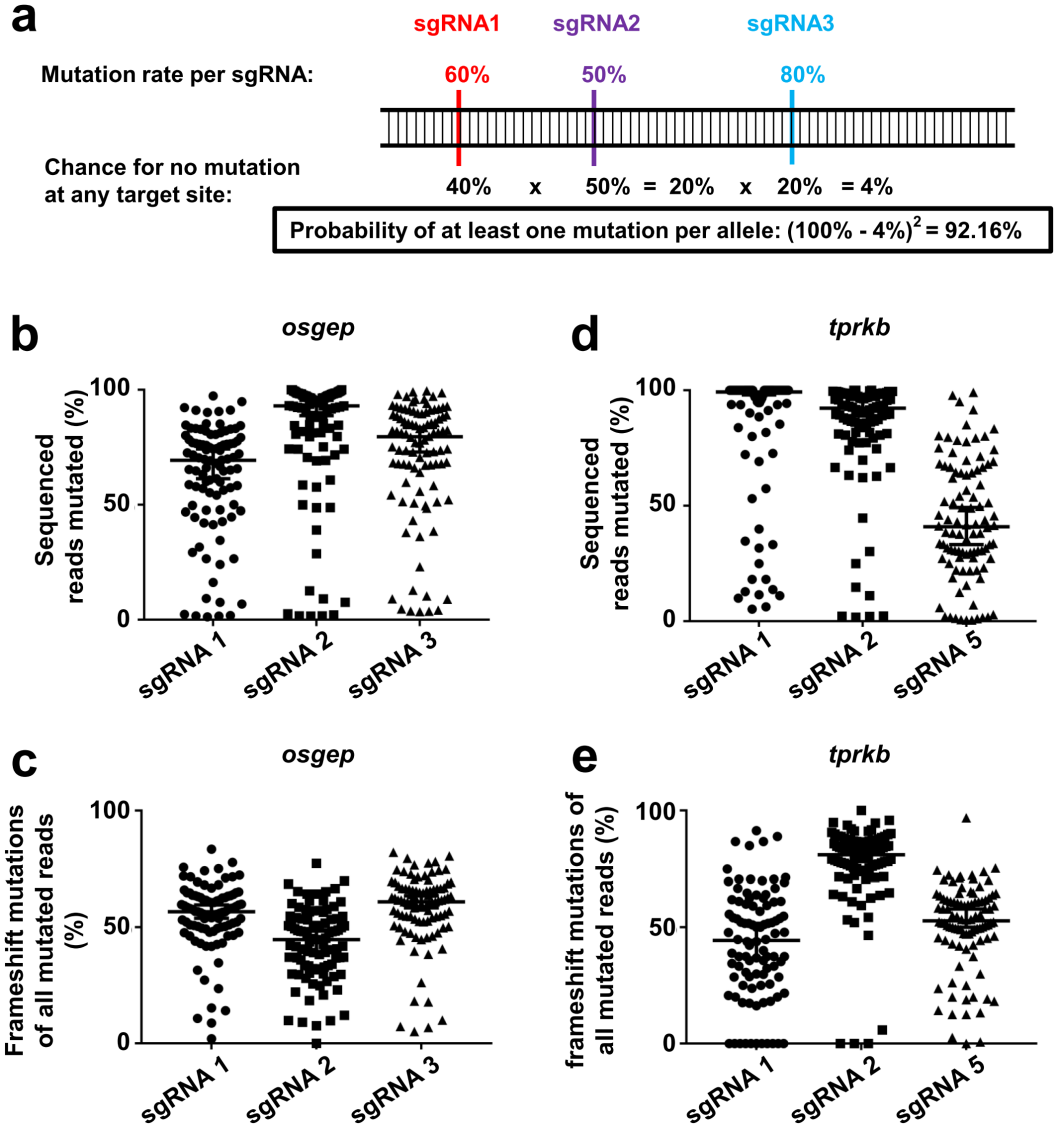
Deep sequencing reveals high mutagenesis rates for acute multi-sgRNA CRISPR/Cas9 KO of *osgep* and *tprkb*. (**a**) Mutation rates per sgRNA (i.e. likelihood of generating at least one mutation on each allele), given hypothetical mutation rates for 3 different sgRNAs. Note that the achieved likelihood for occurrence of at least one mutation per allele is high (92.16%), even though mutation rates for each sgRNA are moderate (50–80%). (**b-e**) Deep sequencing reveals high mutagenesis rates for most sgRNAs and sgRNA dependent frameshift rates. For each gene, deep sequencing data of 96 larvae at 48 hpf were analyzed individually using the tool CRISPResso [24]. (**b**) For *osgep*, median mutagenesis rate was 69.3% for sgRNA1, 93.0% for sgRNA2, and 79.6% for sgRNA3. (**c**) For all mutated *osgep* alleles, the median fractions of frame shifts were 56.7% for sgRNA1, 44.7% for sgRNA2, and 61.0% for sgRNA3. (**d**) For *tprkb*, median mutagenesis rate was 100% for sgRNA1, 92.3% for sgRNA2, and 41.0% for sgRNA5. (**e**) For all mutated *tprkb* alleles, the median fractions of frame shifts were 44.3% for sgRNA1, 81% for sgRNA2, and 52.8% for sgRNA5.

We hypothesized that multiplexed injections of more than two sgRNAs per gene would lead to an increased likelihood for at least one mutation per allele (Fig 1a).

Therefore to achieve increased likelihoods for mutations on both alleles, one cell stage zebrafish embryos were co-injected with a pool of 3 different single guide RNAs (sgRNAs) targeting either *osgep* or *tprkb* (S1 Table) and recombinant Cas9 protein. Embryos injected with a pool of 5 different scrambled sequence sgRNAs and Cas9 protein and uninjected embryos served as controls. For *osgep*, sgRNA1 targeted exon 1, sgRNA2 and sgRNA3 targeted exon 2 (S1A Fig). For *tprkb*, sgRNA1 targeted exon 2, sgRNA2 exon 3, and sgRNA5 exon 1 (S1B Fig).

### Deep sequencing reveals high mutagenesis rates for acute multi-sgRNA CRISPR/Cas9 KO of *osgep* and *tprkb*

To study the degree of genetic mosaicism caused by the multi-plexed injections, we performed amplicon based deep sequencing experiments. Genomic DNA was extracted at 48 hpf from 96 individual embryos per gene that were injected with either *osgep* or *tprkb* targeting sgRNAs. The targeted genomic regions were amplified by an initial targeted PCR. A second barcoding PCR assigned specific barcodes to the combination of individual animal and amplicon. Deep sequencing data were generated using the Illumina MiSeq platform. Sequencing data were analyzed for all specific mutations induced by sgRNAs using the tool CRISPResso [24]. CRISPResso analysis was performed individually per animal and the output was combined for the further calculations. The input parameters included the amplicon reference sequence (derived from assembly Zv9/danRer7), the sgRNA sequence, the coding sequence within the amplicon. Mutations affecting the nucleotides in a window of five nucleotides around the predicted CRISPR/Cas cleavage site (between third and fourth nucleotide upstream the PAM sequence) were considered to be caused by CRISPR/Cas activity. Single nucleotide substitutions (SNS) are typical primary sequencing errors for BridgePCR based Illumina next-generation sequencers [25] and represent PCR errors as well [26]. Additionally, CRISPR/Cas mediated mutagenesis typically causes insertions and deletions. SNS were therefore considered to be not CRISPR/Cas derived and were not included in the calculations.

For *osgep*, sgRNA1 showed a median mutagenesis rate of 69.3%, sgRNA2 a median mutagenesis rate of 93.0%, and sgRNA3 a median mutagenesis rate of 79.6% (Fig 1b). For mutated alleles, the median fractions of frame shifts were 56.7% for sgRNA1, 44.7% for sgRNA2, and 61.0% for sgRNA3, respectively (Fig 1c)

For *tprkb*, sgRNA1 showed a median mutagenesis rate of 100%, sgRNA2 a median mutagenesis rate of 92.3%, and sgRNA5 a median mutagenesis rate of 41.0% (Fig 1d). For mutated alleles, the median fractions of frame shifts were 44.3% for sgRNA1, 81% for sgRNA2, and 52.8% for sgRNA5, respectively (Fig 1e).

**Fig 2:**
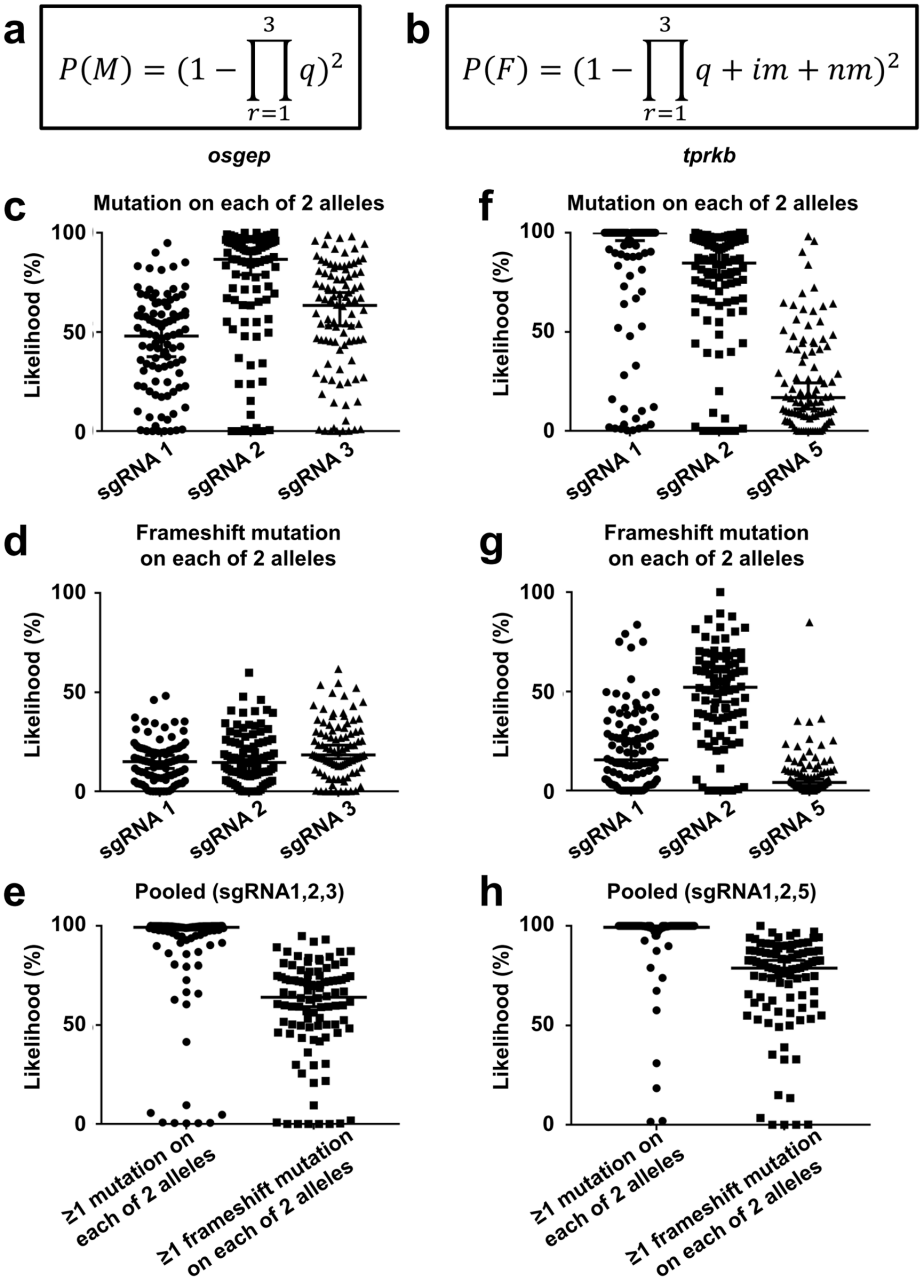
Likelihoods for at least one mutation on each allele and at least one frameshift mutation on each allele are clearly improved in acute multi-sgRNA CRISPR/Cas9 KO of *osgep* and *tprkb*. **(a-b)** The likelihood is shown of generating at least one mutation on each allele *P(M)* (**a**) and of generating at least one frameshift mutation on each allele *P(F)* (**b**), where *M* = at least one mutation on each allele, *F* = at least one frameshift mutation on each allele, *q* = probability of no mutation, *r* = specific sgRNA, *im* = fraction of in-frame mutations of all mutations, *nm* = fraction of non-coding mutations of all mutations. (**c-h**) The likelihoods of at least 1 mutation on each allele and at least 1 frameshift mutation on each allele were calculated based on observed mutagenesis and frameshift rates for 96 individual fish per gene according to the equation in (**a**) and (**b**). (**c**) The individual analysis for *osgep* revealed a median *P(M)* of 48.1% for sgRNA1, 86.6% for sgRNA2, and 63.4% for sgRNA3. (**d**) The median *P(F)* was 15.1% for sgRNA1, 14.6% for sgRNA2, and 18.5% for sgRNA3. (**e**) For the pooled *osgep* sgRNAs, the median *P(M)* was 99.3%, and 64.2% for *P(F)*. (**f**) The individual analysis for *tprkb* revealed a median *P(M)* of 100% for sgRNA1, 84.7% for sgRNA2, and 16.9% for sgRNA5. (**g**) The median *P(F)* was 15.5% for sgRNA1, 52.2% for sgRNA2, and 4.1% for sgRNA5. (**h**) For the pooled *tprkb* sgRNAs, the median *P(M)* was 100%, and 78.8% for *P(F)*.

Based on these data, likelihoods for introduction of at least one mutation on each allele and for at least one frameshift mutation on one allele were calculated according to the equations in Fig 2a and 2b for the sgRNA pools and for each sgRNA individually.

The likelihood to generate at least one mutation on each allele *P(M)* (Fig 2a) is calculated based on the fraction of wildtype alleles of all reads for each individual sgRNA which is the probability of no mutation *q* for each target. The product $(\prod_{r=1}^{3}q)$ of *q* for all three sgRNAs per gene describes the likelihood of having a wildtype allele in all three sgRNA loci and thereby for the entire targeted gene. The term $\left(1-\prod_{r=1}^{3}q\right)$ describes the likelihood of having at least one mutation, looking at a single allele. The square ${\left(1-\prod_{r=1}^{3}q\right)}^{2}$ finally takes into account the presence of two allele per cell and describes the likelihood to generate at least one mutation on each of the two alleles *P(M)* in a diploid organism. The calculation of the likelihood of generating at least one frameshift mutation on each allele *P(F)* (Fig 2b) needs to account for the fractions of inframe mutations *im* and non-coding mutations *nm* in addition to the wildtype alleles *q*. The term (*q* + *im* + *nm*) describes the sum of all non-frameshift alleles for one sgRNA. The product $\left(\prod_{r=1}^{3}q+im+nm\right)$ for all three sgRNAs per gene describes the likelihood of a non-frameshift allele in all three sgRNA loci and thereby for the entire targeted gene and a single allele. In analogy to *P(M)*, ${\left(1-\prod_{r=1}^{3}q+im+nm\right)}^{2}$ finally describes the likelihood of generating at least one frameshift mutation on each allele *P(F)*. The calculations of the likelihoods for the individual sgRNA are described by the simplified equations *P*(*M*) = (1 − *q*)^2^ and *P*(*F*) = (1 − (*q* + *im* + *nm*))^2^.

The individual analysis for *osgep* revealed a median *P(M)* of 48.1% for sgRNA1, 86.6% for sgRNA2, and 63.4% for sgRNA3 (Fig 2c). The median *P(F)* was 15.1% for sgRNA1, 14.6% for sgRNA2, and 18.5% for sgRNA3 (Fig 2d). For the pooled *osgep* sgRNAs, the median *P(M)* was 99.3%, and 64.2% for the median *P(F)*, respectively (Fig 2e).

The individual analysis for *tprkb* revealed a median *P(M)* of 100% for sgRNA1, 84.7% for sgRNA2, and 16.9% for sgRNA5 (Fig 2f). The median *P(F)* was 15.5% for sgRNA1, 52.2% for sgRNA2, and 4.1% for sgRNA5 (Fig 2g). For the pooled *tprkb* sgRNAs, the median *P(M)* was 100%, and 78.8% for the median *P(F)*, respectively (Fig 2h).

Calculations for both sgRNA pools targeting *osgep* or *tprkb* show a significantly higher *P(M)* and *P(F)* than the individual sgRNAs alone, except for *tprkb* sgRNA 1 where *P(M)* was 100% already for this sgRNA individually.

To investigate the relation of *P(F)* and the translation levels of the targeted gene, protein was extracted from 30 pooled zebrafish larvae at 5 dpf per experimental group. Immunoblot and densitometry was performed using Fiji ImageJ [21] and protein levels were compared with scrambled control. The Osgep level was reduced by 61% (S2A Fig) related to a *P(F)* of 61.5%. The Tprkb level was reduced by 51% (S2B Fig) related to a *P(F)* of 78.8%.

Initially, sgRNA selection was performed using the tool CHOPCHOP v1 [16], choosing sgRNA only that had no predicted off-targets. During the execution of this project, the new version CHOPCHOP v2 [27] was released including extended off-target prediction (up to 3 mismatches *versus (vs*.*)* 2 mismatches before). We therefore evaluated the newly identified potential off-targets in the deep sequencing experiment and found that most predicted off-target only showed global mutagenesis rates between 1.1 and 3.1% (S3 Table). The only predicted off-target that shows a relevant mutagenesis rate of 21% in deep sequencing was an off-target for *osgep* sgRNA3 in the gene *col11a2*. However, *P(M)* was only 4.4% and *P(F)* was 0.4% (S4 Table).

All mutations called in the other off-targets were substitutions and therefore not considered to be CRISPR/Cas derived. The off-target 4–3 *tprkb* sgRNA2 features a homozygous noncoding SNP adjacent to the Cas cleavage site that is called as a substitution in 98.1% of all reads.

### Deep sequencing reveals sgRNA specific indel profiles

**Fig 3:**
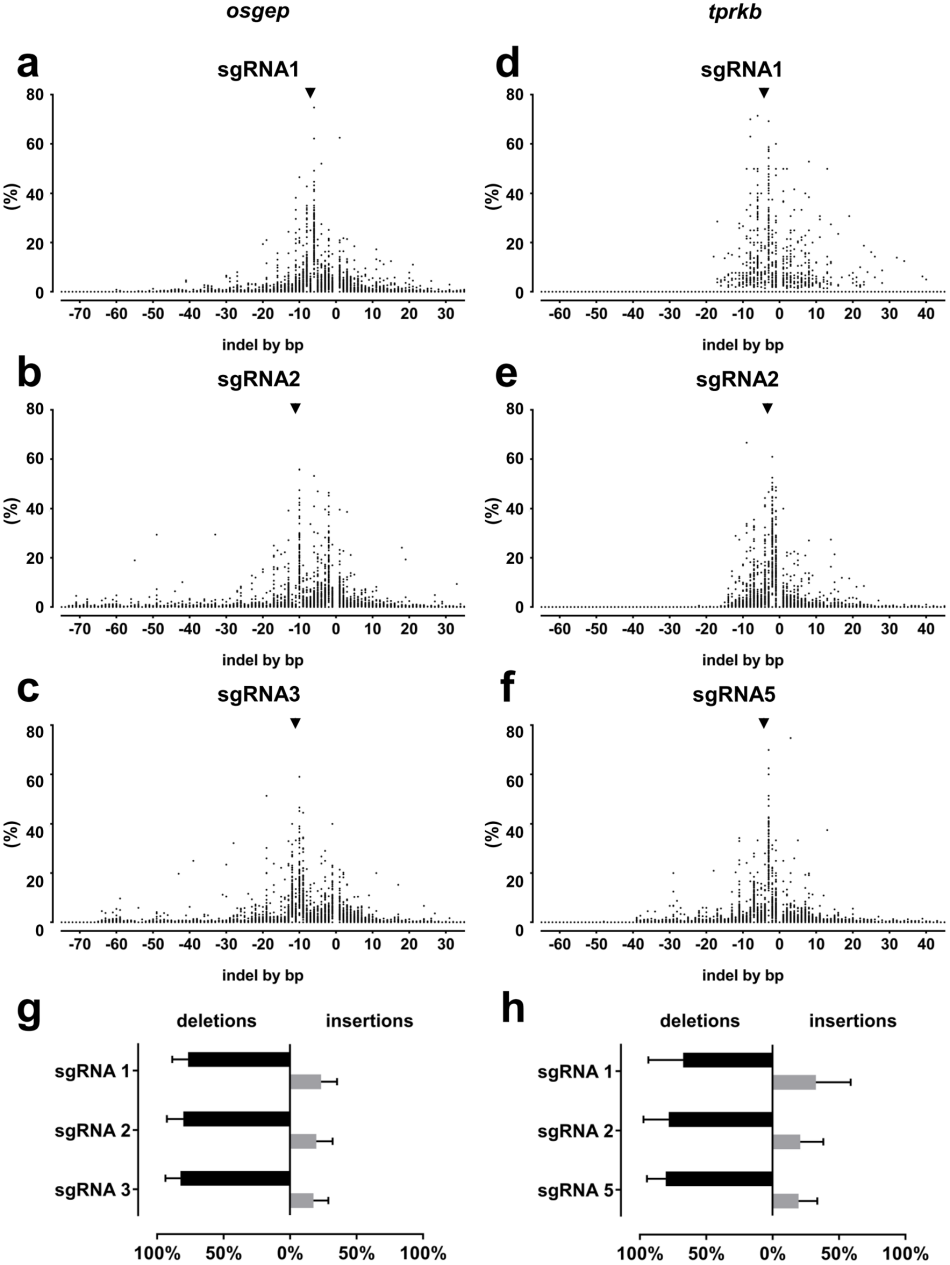
Deep sequencing reveals sgRNA dependent indel profiles. For each gene, deep sequencing data of 96 larvae at 48 hpf were analyzed individually using the tool CRISPResso [24]. (**a-f**) On the X-axis, the graphs show the numbers of base pairs (bp) deleted (negative numbers) or inserted (positive numbers). The Y-axis displays the fraction of the particular indel of all mutated reads per each one of the 96 individual larvae. As many larvae had more than 1 indel, all fractions give the mean frequency in 96 individual larvae. (**a**) For *osgep* sgRNA1, the most frequent deletion was 6 bp in length (23.5%, arrow head), followed by 8 bp (11.1%). The most frequent insertion was 1 bp (6.8%). (**b**) For *osgep* sgRNA2, the most frequent deletion was 10 bp (18.2%, arrow head), followed by 2 bp (16.3%). The most frequent insertion was 1 bp (6.3%). (**c**) For *osgep* sgRNA3, the most frequent deletion was 10 bp (17.1%, arrow head), followed by 12 bp (10.9%). The most frequent insertion was 1 bp (4.7%). *osgep* sgRNA2 and sgRNA3 additionally create larger deletions up to 65 bp together (**b-c**) since they target the same exon. (**d**) For *tprkb* sgRNA1, the most frequent deletion was 3 bp in length (22.9%, arrow head), followed by 6 bp (13.5%). The most frequent insertion had a length of 1 bp (3.9%). (**e**) For *tprkb* sgRNA2, the most frequent deletion was 2 bp (23.8%, arrow head), followed by 1 bp (15.3%). The most frequent insertion was 1 bp (3.1%). (**f**) For *tprkb* sgRNA5, the most frequent deletion was 3 bp (26.9%, arrow head), followed by 2 bp (10.0%). The most frequent insertion was 7 bp (2.6%). (**g**) For *osgep*, deletions accounted for 77.9% of all mutations for sgRNA1, 82.8%for sgRNA2 and 83.6%. for sgRNA3, compared to 22.1% insertion for sgRNA1, 17.2% for sgRNA2 and 16.4% for sgRNA3. (**h**) For *tprkb*, deletions accounted for 73.8% of all mutations for sgRNA1, 83.4%for sgRNA2 and 82.5%. for sgRNA5, compared to 26.2% insertion for sgRNA1, 16.6% for sgRNA2 and 17.5% for sgRNA5.

An ability to predict the rate of sgRNA-induced frameshift mutations in further experiments would help to maximize *P(F)* by selection of sgRNA with high frameshift rates in the screening experiment. We therefore analyzed the sgRNA specific insertion and deletion (indel) profiles for bias towards specific indels. The distribution of different indels is a direct output from CRISPResso. Data from 96 animals per sgRNA were analyzed together as shown in Fig 3 to calculate the median frequencies that are indicated in the following paragraph.

For *osgep* sgRNA1, the most frequent deletion was 6 base pairs (bp) in length (23.5%), followed by 8 bp (11.1%). The most frequent insertion had a length of 1 bp (6.8%) (Fig 3a). For sgRNA2, the most frequent deletion was 10 bp (18.2%), followed by 2 bp (16.3%). The most frequent insertion was 1 bp (6.3%) (Fig 3b). For sgRNA3, the most frequent deletion was 10 bp (17.1%), followed by 12 bp (10.9%). The most frequent insertion was 1 bp (4.7%) (Fig 3c).

In *osgep* exon 2, sgRNA2 and sgRNA3 induced partially larger deletions compared to sgRNA1, possibly due to their proximity. This will likely cause a loss-of-function allele, even when in frame. The introduction of larger deletion by two proximate sgRNAs has been described before [13].

For *tprkb* sgRNA1, the most frequent deletion was 3 bp in length (22.9%), followed by 6 bp (13.5%). The most frequent insertion had a length of 1 bp (3.9%) (Fig 3d). For sgRNA2, the most frequent deletion was 2 bp (23.8%), followed by 1 bp (15.3%). The most frequent insertion was 1 bp (3.1%) (Fig 3e). For sgRNA5, the most frequent deletion was 3 bp (26.9%), followed by 2 bp (10.0%). The most frequent insertion was 7 bp (2.6%) (Fig 3f).

The indel profiles differed between sgRNAs (Fig 3) by indel size and by fraction of the respective indels. In summary, deletions represented a higher fraction of all mutations than insertions (*osgep*: sgRNA1 77.9% *vs*. 22.1%, sgRNA2 82.8% *vs*. 17.2%, sgRNA3 83.6% *vs*. 16.4%, Fig 3g; *tprkb*: sgRNA1 73.8% *vs*. 26.2%, sgRNA2 83.4% *vs*. 16.6%, sgRNA5 82.5% *vs*. 17.5%, Fig 3h) as described before [10, 13].

Notably, one specific deletion can account for up to 27% of all mutations in one specific sgRNA target (e.g. *tprkb* sgRNA5; Fig 3), but with a large variability between the individual animals (0–78%). To assess whether a bias towards frameshift mutations can predicted already for the initial screening, we used the tool Microhomology predictor [28] to calculate the “out-of-frame score” as predictor for the occurrence of frameshift mutations and correlated this score with the observed frameshift rates from our experiment. For an input of 60 nucleotides (30 bp flanking the CRISPR/Cas cleavage site on each side), the predicted out-of-frame-score did not correlate with the observed frameshift rates (R^2^ = 0.0299, S3 Fig), supporting the necessity of an initial screening experiment.

### Acute multi-sgRNA CRISPR/Cas9 KO reproduces the survival phenotype of stable KO lines for *osgep* and *tprkb*

Our deep sequencing data analysis showed high values for *P(M)* and *P(F)* for the acute multi-sgRNA KO, and an impact on protein levels as well. We therefore hypothesized that these genomic and translational changes would cause a phenotype similarly to what we described previously for stable *osgep* and *tprkb* KO [15]. We have shown previously that stable KO zebrafish larvae, for both *osgep* and *tprkb*, established reduced survival [15]. We therefore investigated whether the acute multi-sgRNA KO would show reduced survival as well.

**Fig 4:**
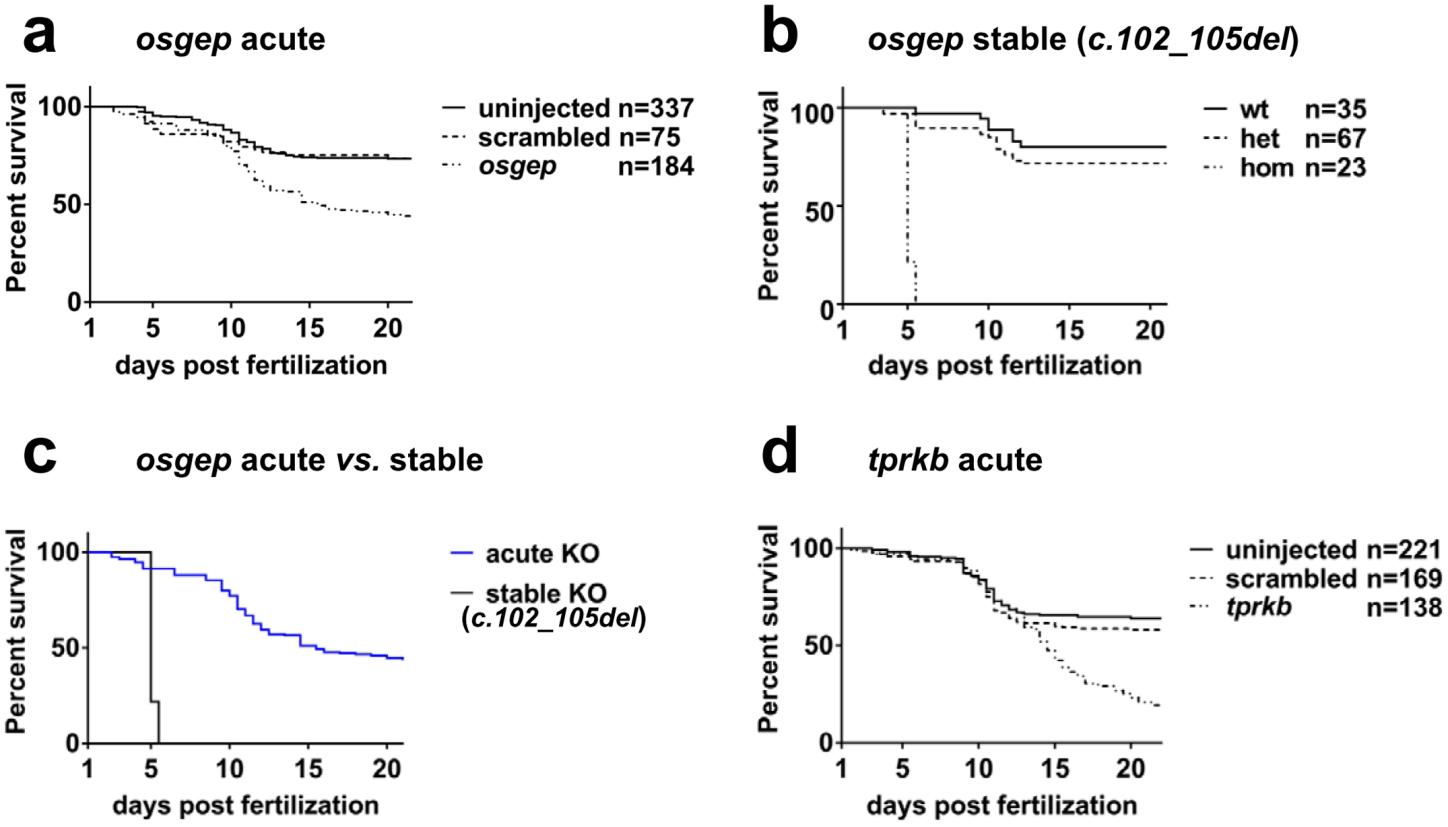
Acute multi-sgRNA CRISPR/Cas9 KO and stable KO lines for *osgep* and *tprkb* demonstrate reduced survival. In order to generate survival curves, larvae were monitored twice a day for 22 days and transferred to rotifer feeding solution at 8 dpf. Tracing started at 24 hpf. (**a**) The acute multi-sgRNA KO for *osgep* shows an increased death rate as of 10 dpf with a survival of 42.9% at 22 dpf compared to scrambled and uninjected control with both survival of 73.4% at 22 dpf (P<0.0001). (**b**) In the stable *osgep* KO line with the truncating mutation *c*.*102_105del*, homozygous larvae show complete mortality by 5 dpf, whereas 71% of the heterozygous and 80% of the wildtype larvae survive until the end of the observation at 21 dpf. No significant difference in survival was found between heterozygous and wildtype larvae (P = 0.3269). (**c**) For *osgep*, homozygous stable KO larvae show a significantly increased and earlier mortality compared to acute KO larvae (P<0.0001). (**d**) The acute multi-sgRNA KO for *tprkb* shows an increased death rate as of 12 dpf with a survival of 19.4% at 22 dpf compared to scrambled (58.0%) and uninjected control (63.8%) at 22 dpf (P<0.0001).

For *osgep*, the survival rate in the multi-sgRNA KO was significantly lower with 42% at 22 dpf compared to 73.4% for the uninjected and 73.4% for scrambled control groups (*X*^*2*^ = 54.83, P<0.0001) (Fig 4a). Survival rates were not significantly different between scambled and uninjected control (*X*^*2*^ = 0.01562, P = 0.9005).

We here report an additional allele for the stable *osgep* KO that is different from the one we reported previously [15] to allow for direct comparison between acute and stable phenotypes. We found that all homozygous larvae of the *osgep* KO line *c*.*102_105del* die between 3 dpf and 5 dpf (Fig 4b) (*X*^*2*^ = 113.8, P<0.0001). Wildtype and heterozygous control larvae do not show a significant difference in survival (*X*^*2*^ = 0.9611, P = 0.3269).

A comparison between acute KO larvae and homozygous stable KO larvae for *osgep* identified a significantly increased survivability for acute KO over stable KO (Fig 4c) (*X*^*2*^ = 102.7, P<0.0001; median survival 15 *vs*. 5 dpf).

For *tprkb*, the survival rate in the multi-sgRNA KO was significant lower with 19.4% compared to 63.8% for uninjected and 58.0% for scrambled control (*X*^*2*^ = 49.47, P<0.0001) (Fig 4d). Survival rates were not significantly different between scrambled and uninjected control (*X*^*2*^ = 1.349, P = 0.2455).

When compared to the homozygous stable KO larvae for *tprkb* published previously [15], we identified a significantly identified a significantly increased survivability for acute KO over stable KO (*X*^*2*^ = 44.81, P<0.0001; median survival 14 vs. 11 dpf).

### Microcephaly phenotype in zebrafish

We have shown previously that stable KO zebrafish larvae, for both *osgep* and *tprkb*, display a distinct microcephaly phenotype [15], recapitulating the human neuronal phenotype for mutations in the human orthologues. We therefore investigated whether the acute multi-sgRNA KO would reproduce this phenotype as well. Furthermore, we here report an additional stable KO allele for each of the two genes *osgep* and *tprkb* that are different from the one we reported previously [15].

**Fig 5:**
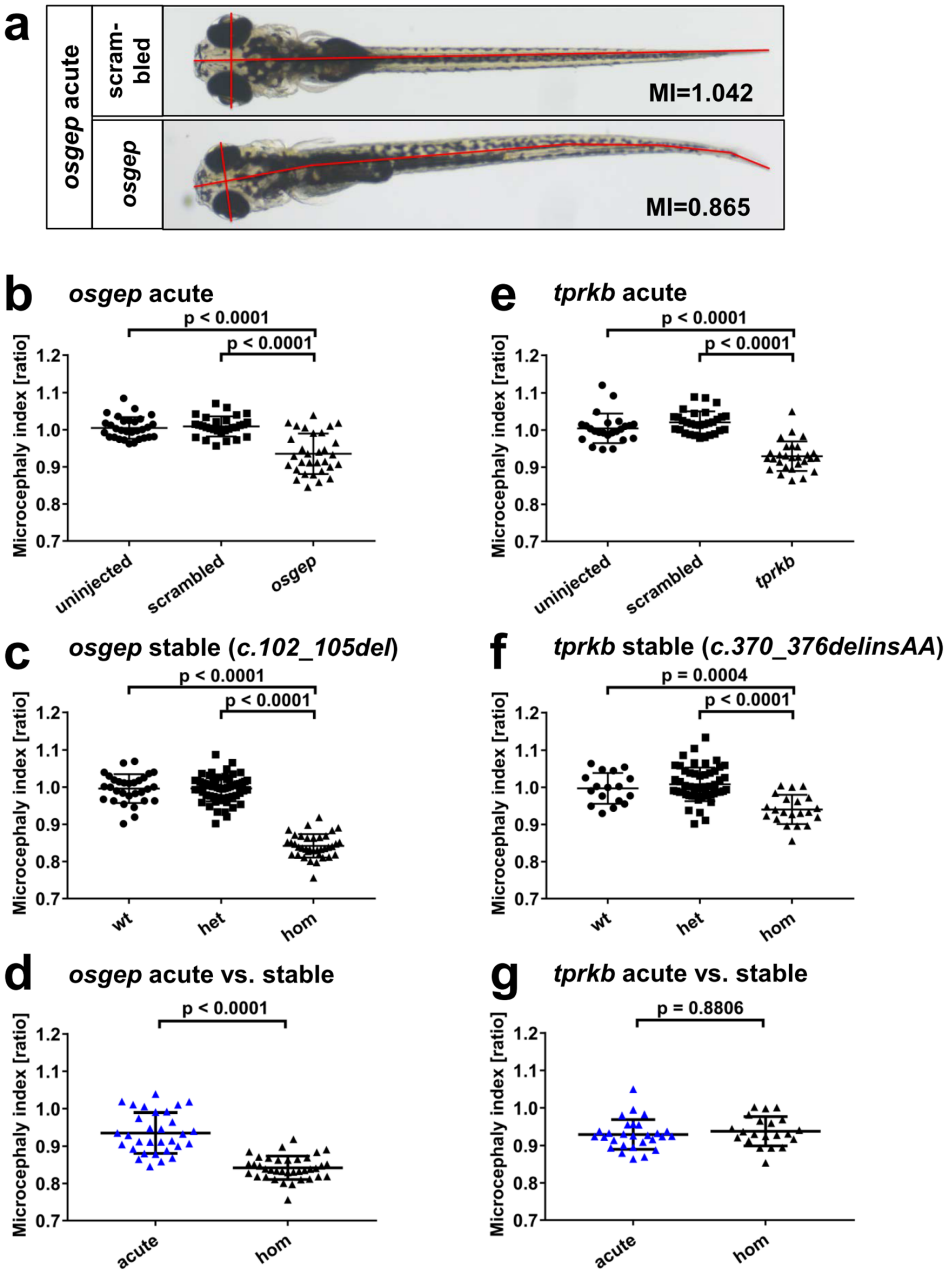
Acute multi-sgRNA CRISPR/Cas9 knockout reproduces the microcephaly phenotype of stable *osgep* and *tprkb* KO lines. Larvae were imaged from a dorsal view. Head diameter to total body length was calculated. This ratio was normalized by the mean of the uninjected/wildtype control and defined as “microcephaly index” (MI). MI was determined at 6 dpf except for the stable KO line osgep c.102_105del at 4 dpf due to early lethality (see Fig 4a). (**a**) Representative phenotypes of a larva that was injected with the *osgep* targeting multi-sgRNA pool compared to a scrambled control larva. The red lines display the typical axes used for the measurements. (**b**) For *osgep*, the acute multi-sgRNA KO showed a significant microcephaly compared to uninjected and scrambled control. (**c**) The microcephaly phenotype is recapitulated in the homozygous larvae of the stable KO line *osgep c*.*102_105del* compared to wildtype and heterozygous clutch mates. (**d**) For *osgep*, MI is significantly higher in acute KO larvae compared to homozygous stable KO larvae. (**e**) For *tprkb*, the acute multi-sgRNA KO showed a significant microcephaly compared to uninjected and scrambled control. (**f**) The microcephaly is recapitulated in the homozygous larvae of the stable KO line *tprkb* c.370_376delinsAA compared to wildtype and heterozygous clutch mates. (**g**) For *tprkb*, no significant difference in MI was found for acute KO larvae compared to homozygous stable KO larvae.

To assess microcephaly in zebrafish larvae, we collected dorsal images at 6 dpf and calculated head diameter to body length ratio to define a microcephaly index. Both parameters were obtained using Fiji ImageJ [21]. Then to compare between stable and acute lines we normalized each group by dividing by the mean of the respective uninjected or wildtype group (Fig 5a). Due to early lethality of homozygous *osgep* c102_105del larvae by 5dpf, microcephaly indices were determined at 4 dpf.

Acute multi-sgRNA KO of *osgep* in zebrafish larvae recapitulated the microcephaly phenotype that we found previously in stable *osgep* KO (Fig 5b). Acute *osgep* KO larvae had a significantly smaller median microcephaly index of 0.9291 *vs*. 1.006 in scrambled control (P<0.0001). The microcephaly was also observed in the newly reported stable *osgep* KO line c.102_105del at 4 dpf (Fig 5c). The median microcephaly index was significantly lower in homozygous larvae with 0.8384 *vs*. 1.001 in heterozygous larvae (P<0.0001).

We found a significantly higher microcephaly index in the acute *osgep* KO larvae in comparison to homozygous stable *osgep* KO larvae. (Fig 5d) (P<0.0001).

Acute multi-sgRNA KO of *tprkb* in zebrafish larvae recapitulated the microcephaly phenotype that we found previously in stable *tprkb* KO (Fig 5e). Acute *tprkb* KO larvae had a significantly lower median microcephaly index of 0.9256 *vs*. 1.018 in scrambled control (P<0.0001). The microcephaly phenotype was also observed in the newly reported stable *tprkb* KO line *c*.*370_376delinsAA* (Fig 5f). The median microcephaly index was significantly lower in homozygous larvae with 0.9332 *vs*. 1.001 in heterozygous larvae (P<0.0001).

No significant difference in microcephaly index was determined between the acute *tprkb* KO larvae and homozygous stable *tprkb* KO larvae. (Fig 5g, P = 0.8806).

## Discussion

In this work, we apply an acute multi sgRNA KO approach for two exemplar genes, *osgep* and *tprkb*, to increase the degree of mutagenesis and thereby the frequency of the phenotype in the injected F0 generation. In contrast to previous applications of multiplexed sgRNA that were primarily used for sgRNA testing [13, 17] or screening [12] by targeting multiple genes, we separately target individual genes, quantify the degree of mutagenesis individually for each animal by deep sequencing and provide a mathematical model to describe the probable loss-of-function.

We demonstrated that the use of three pooled different sgRNA targeting the same gene results in a nearly complete mutagenesis of the respective gene in the whole zebrafish embryo. In contrast, mutagenesis rates are lower for most individual sgRNAs. Additionally, the likelihood for carrying at least one frameshift mutation on each allele is significantly improved compared to each individual sgRNA (Fig 2). However, we found that the acute multi-sgRNA KO for *osgep* and *tprkb* still showed a less prominent phenotype than the stable KO lines for *osgep* and *tprkb* (Figs 4 and 5, [15]). The difference in survival and microcephaly phenotype between acute multi-sgRNA KO and stable KO lines might be explained by the existence of a full gene KO by a homozygous truncating mutation in the stable line in contrast to a mosaic genotype in the acute multi-sgRNA KO with a mix of truncating and non-truncating mutations (Figs 1 and 2). The mosaic larvae will therefore still express some functional protein in a subset of cells. This is clearly the limitation of the acute multi-sgRNA approach compared to a stable KO line when a maximal KO effect is desired. However, if functional domains of the targeted protein are known, these can be targeted in this approach to maximize the KO effect, since in frame indel mutations might affect these domains as well [29].

When studying genes that are essential for early development, but also play a role in later larval growth, a full KO may lead to early death and prevent the investigation of these genes during later larval stages, as seen for example in the stable *osgep* KO larvae ([15], Fig 4b). Thus, the weaker phenotype with delayed lethality in the mosaic larvae might be even desirable in certain experimental settings.

In contrast to breeding of heterozygous zebrafish KO line that produce embryo clutches in a Mendelian ratio, where only 25% of the embryos have the desired genotype, the acute multi-sgRNA KO provides one with a tool to generate a large cohort of mosaic KO embryos in a short amount of time. However, similar to MO experiments, effect size, toxicity and off-target effects are potential issues in acute CRISPR approaches. Therefore, careful experimentation including the use of scrambled sgRNA injections to exclude unspecific injection effects, the newest prediction algorithms for minimizing off-targets [27], and our mathematical model for estimating the effect size all increase the plausibility of such experiments.

Interestingly, deep sequencing data revealed sgRNA specific indel profiles in which the deletions introduced are not randomly distributed, but seem to favor a particular length of deletion dependent on the sgRNA or targeted locus as described before [17]. Notably, one specific deletion can account for up to 27% of all mutations (mean of 96 individual larvae) in one specific locus (e.g. *tprkb* sgRNA5; Fig 3), but with a large variability between the individual animals (0–78%).

One reason for locus specific editing is microhomology-mediated end joining (MMEJ), also known as alternative non-homologues end joining (NHEJ), a process that is mediated by base pairing between microhomologues sequences of 2–25 nucleotides [30, 31]. MMEJ in zebrafish is dependent on DNA ligase 3 (*lig3*) whereas the canonical NHEJ depends on DNA ligase 4 (*lig4)* [32]. MMEJ is considered to be error prone and highly mutagenic, contributing to genome instability in cancer [33–35]. In addition, MMEJ is biased towards locus specific mutations [36]. Given a high MMEJ activity in early zebrafish development in parallel to canonical NHEJ [32], this mechanism is likely to explain the locus specific preferences for certain indels found in this study. Although tools exist to predict MMEJ bias for frameshift mutations [28], we do not find a positive correlation between prediction and observed frameshift rates. In contrast, empirically determined indel profiles as obtained in this study can be used to predict allelic outcomes for future experiments, and allow choosing specific sgRNAs to enrich for desired mutations.

In the acute multi-sgRNA approach, the reduction of protein level corresponded to the percentage of potential nonsense mutations for *osgep* (61% vs. 64%), but not for *tprkb* (41% vs. 78%). One would expect that a higher rate of nonsense mutations would also lead to a more significant reduction in protein amount if influenced only by the intact gene copy numbers in the whole animal. These data suggest a longer half-life of the maternal *tprkb* mRNA, the protein derived from this RNA, or both, all in contrast to *osgep*. Previous studies have described that the length of the 3’untranslated region (UTR) is a factor that determines maternal mRNA stability in zebrafish during the maternal-to-zygotic transition [37]. In this study, *tprkb* mRNA features a 3’ UTR of 634 nucleotides versus 92 nucleotides in the *osgep* mRNA which might lead to an earlier degradation and depletion of maternal *osgep* mRNA in homozygous *osgep* KO larvae compared to maternal *tprkb* mRNA in homozygous *tprkb* KO larvae. The presence of maternal mRNA, that is transmitted from the heterozygous mother to the homozygous embryo [3], might be also one of the factors that caused the poor correlation between MO morphant and mutant phenotypes [4]. Translation-blocking MOs target all mRNA, whereas a recessive gene KO will only affect the mRNA newly transcribed from the mutated zygotic genome. Especially when targeting genes where maternal mRNA is stable over a long time and the derived protein has a long half-life, one might miss the mutant phenotype, at least in the observation period of up to 7 dpf that is typical for MO experiments. Thus, observation periods may have to be extended when using CRISPR/Cas9 instead.

To overcome the influence of maternal mRNA, splice-targeting MOs or the recent discovery of the RNA targeting CRISPR type VI protein C2c2 might offer a suitable new approach that could be adapted for zebrafish [38]. Future studies could validate in both cases, if injections in embryos of stable KO lines would only lead to a depletion of the maternal mRNA, unmask an effect of this mRNA, and lead to an early phenotype.

In conclusion, this study provides further evidence that multiplexed CRISPR/Cas injections can be a reliable KO technique with little off-target effects, and is the first to provide a mathematical model to quantitatively describe the degree of deleteriousness in such application.

## Accession numbers

*D*.*rerio osgep* cDNA (NM_001017751), *D*.*rerio tprkb* cDNA (NM_001007373).

## Supporting information

S1 Fig Genetic map of *osgep* and *tprkb*. Genetic map of the zebrafish genes *osgep* (**A**) and *tprkb* (**B**), respectively. Yellow arrows represent introns. Green arrows represent exons. Blue arrows represent positions of sgRNAs. Red bars represent PAM sequences. Three different sgRNAs per gene were selected using the CHOPCHOP v1 web tool (15). sgRNAs were chosen according to the ranking by CHOPCHOP, avoiding overlaps in protospacer and PAM sequences and preferentially targeting early exons.
(TIF)

S2 Fig Acute multi-sgRNA CRISPR/Cas9 knockout of the zebrafish genes *osgep* and *tprkb* leads to a significant reduction protein levels. Immunoblot on 30 pooled larvae at 6 dpf shows a reduction of (**A, C**) osgep and (**B, D**) tprkb compared each to scrambled and uninjected control. Densitometry shows a relative reduction in protein levels to 39% for osgep (**C**) and 59% for tprkb (**D**), each compared to scrambled control.
(TIF)

S3 Fig Predicted frameshift scores do not correlate with observed frameshift rates. X-axis displays out-of-frame-score for each sgRNA as prediction for frameshift rates according to the tool Microhomology predictor (Bae S, *Nat*. *Methods*, 11:705, 2014). Y-axis displays median frameshift rates that were found for each sgRNA by deep sequencing. No significant correlation was found for both parameters (R^2^ = 0.0299, P = 0.74).
(TIF)

S1 Table sgRNA sequences targeting zebrafish KEOPS genes. (XLSX)

S2 Table Primer sequences for deep sequencing. (XLSX)

S3 Table Mutagenesis off-targets. (XLSX)

S4 Table Calculation likelihoods for off-target *col11A2*. (XLSX)

S5 Table Mutagenesis calculations. Raw data of Figs 1 and 2.
(XLSX)

S6 Table Indel profiles. Raw data of Fig 3.
(XLSX)

S7 Table Survival data. Raw data of Fig 4.
(XLSX)

S8 Table Microcephaly measurements. Raw data of Fig 5.
(XLSX)

S9 Table Microhomology prediction. Raw data of S3 Fig.
(XLSX)
