# Supplementary figures and images for: Acute multi-sgRNA knockdown of KEOPS complex genes reproduces the microcephaly phenotype of the stable knockout zebrafish model
Source: PLoS One. 2018 Jan 18;13(1):e0191503. doi: 10.1371/journal.pone.0191503 (PMC5773193; doi:10.1371/journal.pone.0191503)

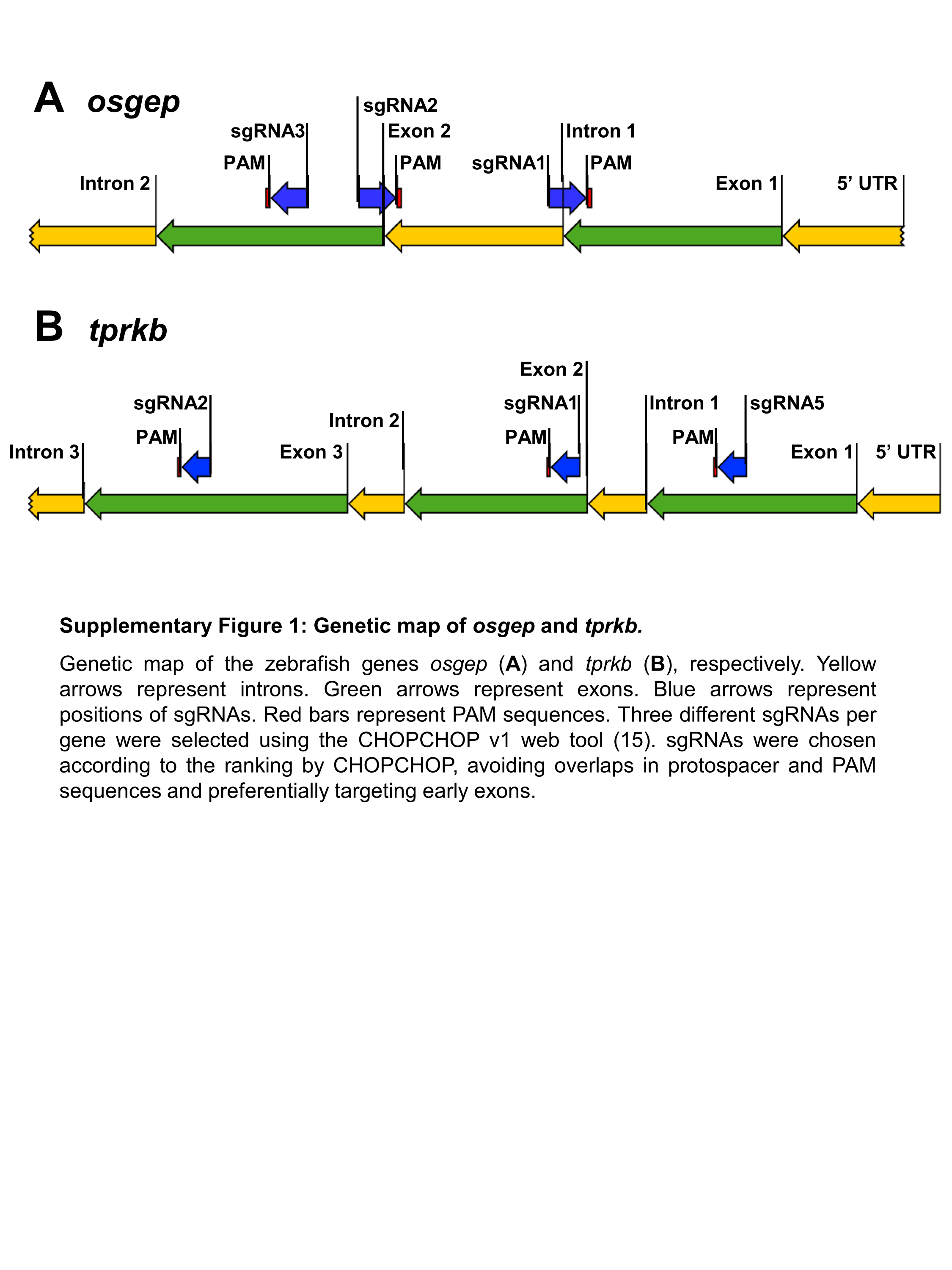

Supplement: S1 Fig — Genetic map of the zebrafish genes osgep (A) and tprkb (B), respectively. Yellow arrows represent introns. Green arrows represent exons. Blue arrows represent positions of sgRNAs. Red bars represent PAM sequences. Three different sgRNAs per gene were selected using the CHOPCHOP v1 web tool (15). sgRNAs were chosen according to the ranking by CHOPCHOP, avoiding overlaps in protospacer and PAM sequences and preferentially targeting early exons. (TIF) [file pone.0191503.s001.tif]

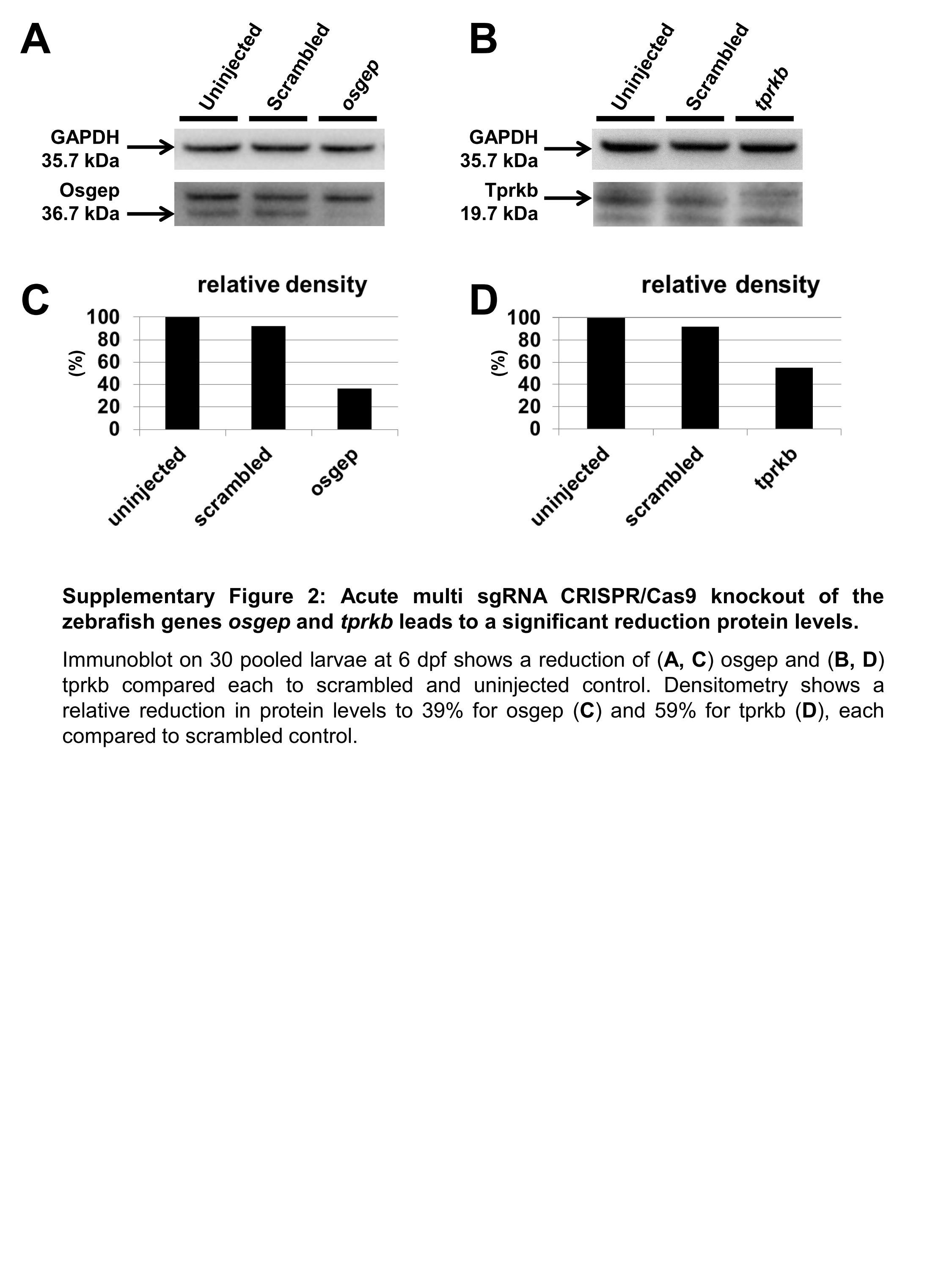

Supplement: S2 Fig — Immunoblot on 30 pooled larvae at 6 dpf shows a reduction of (A, C) osgep and (B, D) tprkb compared each to scrambled and uninjected control. Densitometry shows a relative reduction in protein levels to 39% for osgep (C) and 59% for tprkb (D), each compared to scrambled control. (TIF) [file pone.0191503.s002.tif]

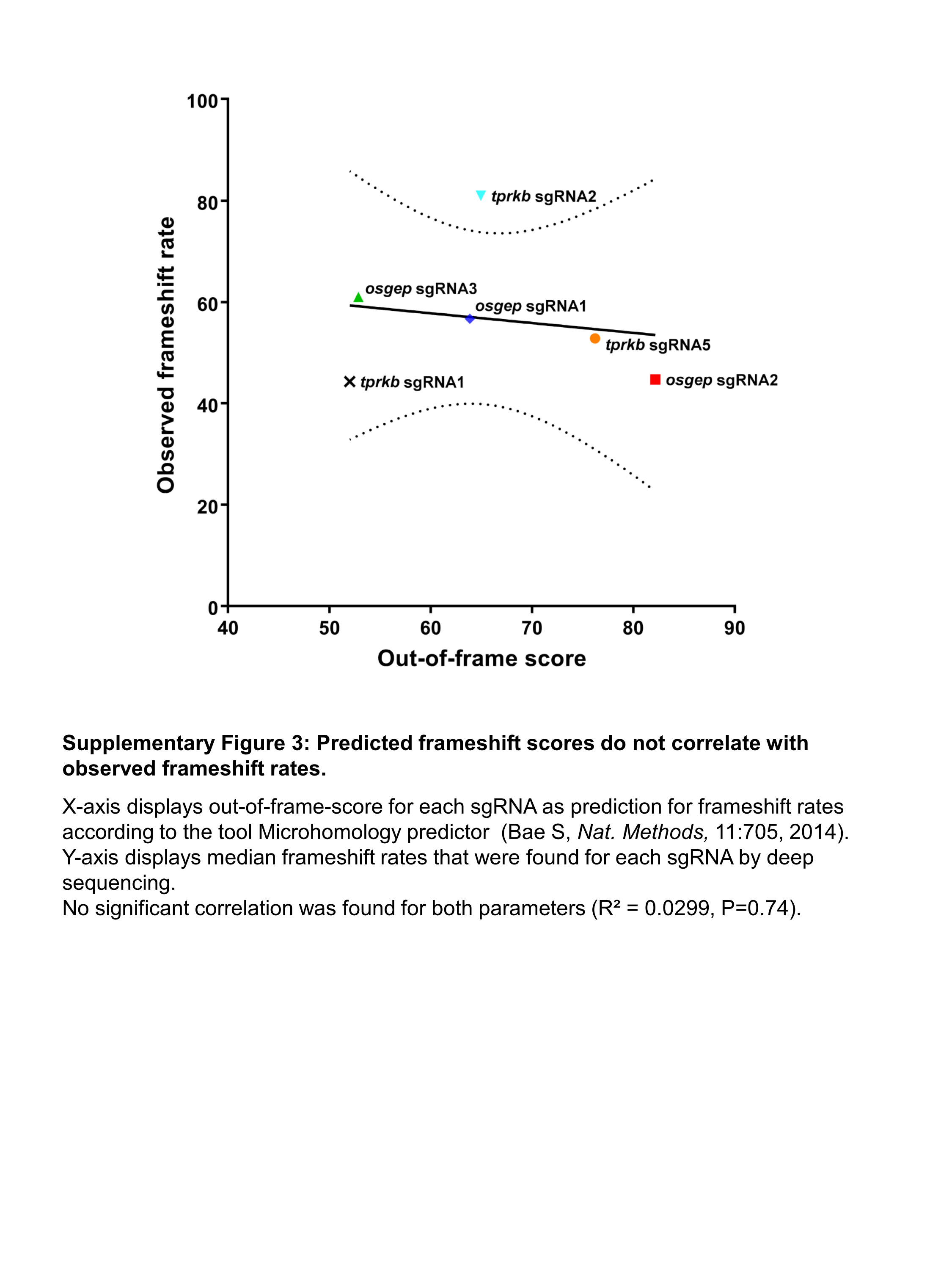

Supplement: S3 Fig — X-axis displays out-of-frame-score for each sgRNA as prediction for frameshift rates according to the tool Microhomology predictor (Bae S, Nat. Methods, 11:705, 2014). Y-axis displays median frameshift rates that were found for each sgRNA by deep sequencing. No significant correlation was found for both parameters (R2 = 0.0299, P = 0.74). (TIF) [file pone.0191503.s003.tif]
